# Malawi polio outbreak: What next?

**DOI:** 10.1002/puh2.19

**Published:** 2022-09-05

**Authors:** Isabel Kazanga‐Chiumia, Symon Fidelis Nayupe, Betty Kazanga, Steven Munharo, Parth Patel, Phatisan Bango Jassi, John Phuka

**Affiliations:** ^1^ School of Global and Public Health Kamuzu University of Health Sciences Lilongwe Malawi; ^2^ Private Clinic Kamuzu University of Health Sciences Blantyre Malawi; ^3^ IRD, INSERM Aix Marseille Univ, SESSTIM, ISSPAM Marseille France; ^4^ Chikwawa Diocese St Montfort Mission Hospital Chikwawa Malawi; ^5^ Atlas Medical Center Blantyre Malawi; ^6^ World Health Organization Lilongwe Malawi

**Keywords:** Malawi, outbreak, polio, response

## Abstract

Since, the last polio case was in 1992, health authorities in Malawi declared an outbreak of wild poliovirus type 1 (WPV1) on 17 February 2022. A 3‐year‐old girl was diagnosed with WPV1 in the country's capital, Lilongwe, after getting paralyzed by the virus in November 2021. The re‐emergence of polio presents a new public health challenge that Malawi must respond to avoid the further spread of the virus within and outside the country. With an ongoing Coronavirus disease (Covid‐19) pandemic, responding to polio could be a challenge as the healthcare system is already challenged with responding to the pandemic. Frequent cross‐border movement in the region also poses another challenge, particularly in the background of poor vaccination coverage and poor‐quality vaccines. We recommend the development of a risk assessment plan that will guide in implementing vaccination campaigns. Additionally, polio surveillance programmes must be put in place with strong political, stakeholder and community engagement.

## INTRODUCTION

Health authorities in Malawi declared an outbreak of wild poliovirus type 1 (WPV1) on 17 February 2022, after a 30‐year polio‐free period [1]. A 3‐year‐old girl was diagnosed with WPV1 in the country's capital, Lilongwe, after she was paralyzed by the virus in November 2021. Laboratory analysis results showed that the child was infected by a strain of poliovirus that was genetically linked to a strain detected in Pakistan's Sindh province in October 2019 [2].

Polio is a highly infectious disease caused by a virus that is transmitted from one person to another [1]. It spreads through the fecal‐oral route and contaminated water or food. After multiplying in the intestines, the polio virus invades the nervous system and can lead to irreversible total paralysis within hours. Death occurs due to failure of affected breathing muscles and it mainly affects children under 5 years of age. Since, polio spreads quickly, a single detection of the virus is considered an outbreak [3]. While there is no cure for polio, the disease can be prevented through the administration of the polio vaccine, given multiple times to protect a child for life [1].

The World Health Organisation (WHO) reported that the new case in Malawi is the first case of WPV1 in Africa in over half a decade [1]. In August 2020, Africa was declared free of indigenous wild polio after eliminating all forms of wild polio from the region. Globally, since 1988 the number of polio cases has fallen by more than 99%, due to the Global Polio Eradication Initiative (GPEI) [2]. However, persistent pockets of polio transmission in Afghanistan and Pakistan have continued to put unvaccinated children everywhere at risk [1]. Previous outbreaks in Africa were mostly due to vaccine‐derived polio, which occurs in areas of low immunisation when the live but attenuated virus in the oral polio vaccine reverts and regains its ability to paralyze and spread [4].

The GPEI says the re‐emergence of polio in Malawi is the latest setback for the global campaign to end polio once and for all [3]. The identification of WPV1 in Malawi and its continued existence in the world's two remaining endemic countries, Pakistan and Afghanistan, is a significant concern that underscores the importance of surveillance and prioritizing polio immunisation activities at national, regional and global levels [2]. Furthermore, the declaration of the outbreak in Malawi raises the question of whether the country and Africa, in general, will lose or maintain the polio‐free certification. Despite Malawi declaring an outbreak, the WHO states that the polio‐free status remains for Africa, particularly because the case is imported [1].

## MALAWI'S POLIO SITUATION AND CHALLENGES

Currently, Malawi is faced with a major challenge to prevent the spread of the virus locally and beyond its borders, particularly because of frequent migration in the region [4]. Another concern is that the polio outbreak has been declared while the country is still battling the COVID‐19 pandemic, thereby putting pressure on the already overstretched health system. It has also been reported that the quality of polio surveillance in Malawi has slipped during the COVID‐19 pandemic [3], further raising the need to boost surveillance.

## RECOMMENDATIONS ON MALAWI'S RESPONSE TO THE OUTBREAK

Previous Malawi polio eradication efforts have been due to massive vaccination of children and the global polio eradication initiative has a history of managing imported outbreaks quickly with rapid vaccination campaigns [3, 4]. The goal is to stop the spread within 6 months before the virus becomes entrenched [3]. Evidence shows that basic strategies to eradicate polio are: attaining high routine coverage with at least three doses of OPV; conducting immunisation campaigns; and establishing a sensitive surveillance system of AFP to track WPV1 circulation [5]. Therefore, permanent elimination of the poliovirus can only be achieved by maintaining a high rate of population immunity [5]. Thus, Malawi should focus on strengthening the immunisation programme and quality of the surveillance system to stop transmission, eliminate the virus, and ensure early detection and effective response. The Ministry of Health and key stakeholders should immediately develop a thorough risk assessment plan to ascertain the risk that Malawi is in. High risk will demand large‐scale control programmes with nationwide vaccinations of children under the age of 5 years to curb the spread.

Additionally, the global polio eradication strategy 2022–2026 also emphasizes the need to generate greater political will, strengthen stakeholder involvement, improve community engagement to overcome vaccine hesitancy and increase uptake, and integrate polio immunisation with immunisation activities for other diseases such as COVID‐19 [6]. Studying factors that influence immunisation rates in the country to carefully plan a robust immunisation initiative is important [5]. This is crucial considering the devastating impact COVID‐19 misinformation has had on immunisation programs in Malawi. Additionally, polio eradication strategies should be tailor‐made for the Malawian context targeting low polio immunisation groups to equitably reach immunisation gaps widened over the years.

## CONCLUSION

The current polio case is a public health concern that should be addressed to prevent the spread of the virus in and outside Malawi. The re‐emergence of the virus should trigger an evaluation of the entire polio management and response plan aimed at eradicating the disease in the country and region once and for all. For the current case, risk assessment followed by mass vaccinations, disease surveillance and polio awareness campaigns need to be implemented in Malawi and extended to the borders of Zambia, Tanzania and Mozambique.

## AUTHOR CONTRIBUTIONS

Isabel Kazanga‐Chiumia and Parth Patel conceptualized the idea of the paper. Symon Fidelis Nayupe, Betty Kazanga, Steven Munharo, Phatisan Bango Jassi and John Phuka contributed to the gathering of data. All authors contributed to the analysis of the data and the revision iterations. Final rewriting and revision were done by Isabel Kazanga‐Chiumia, Symon Fidelis Nayupe, Steven Munharo and Betty Kazanga. All co‐authors agreed to the final draft of the paper.

## CONFLICT OF INTEREST

The authors declare no competing interest.

## FUNDING

There is no funding for the development of this paper.

## ETHICAL APPROVAL

There is no need for ethical approval.

## Data Availability

No data base or primary data was used in writting the pape.
